# Supplementary material for: Predictors of obesity among school-age children in Debre Berhan City, Ethiopia
Source: PLOS Glob Public Health. 2023 Sep 8;3(9):e0001895. doi: 10.1371/journal.pgph.0001895 (PMC10490846; doi:10.1371/journal.pgph.0001895)
Supplement: S1 File — (DOCX) [file pgph.0001895.s002.docx]

**Annex. English version questionary for predictors of obesity among school age children in Debre-berhan city Amhara Ethiopia, 2022.**

**PART I**: **Questionnaire for obesity Risk Factor Surveillance to be filled by student family**

Participant Identification Number___________

Kebele --------------------------------------- Name of village ------------------------------

| **Socio-demographic information** | | Response | | Remark | |
| --- | --- | --- | --- | --- | --- |
| Q101 | Sex | 1. Female 2. Male | |  | |
| Q102 | Age of child’s mother | ---------------------- | |  | |
| Q103 | Educational status of mother | 1. Can’t read and write 2. Able to read &write 3. Primary school 4. Secondary school 5. College and above | |  | |
| Q104 | Educational status of father | 1. Can’t read and write 2. Able to read &write 3. Primary school 4. Secondary school 5. College and above | |  | |
| Q105 | Occupation of the Mothers | 1. House wife  2. Government Employee  3. Private Business  4. daily laborer | |  | |
| Q106 | Occupation of the Fathers | 1. Government employee  2. Private Business  3.daily laborer | |  | |
| Q107 | Estimated monthly income | **------------------------** | |  | |
| Q108 | Family size | **-------------------------** | |  | |
| Q109 | Is there vehicle to transport family from place to place? | 1.yes 2.no | |  | |
| Q110 | Child birth weight | ------------------------ | |  | |
| Q111 | Complete immunization of the child | 1.Yes 2.No | |  | |
| **Parental style and characteristics** | | | | | |
| Q201 | How often does your family eat at least one meal together each day? | | 1. Never  2. 1-2 times per week  3.3-4 times per week  4.daily | |  |
| Q202 | How often in a week does your family eat fruits and/or vegetables with your main meal? | | 1. Never  2.1-2 times per week  3.3-4 times per week  4. daily | |  |
| Q203 | Does your family have adequate space for the children to play? | | 1. Yes 2. No | |  |
| Q204 | Does your family encourage the child to be physically active or play sports? | | 1. Yes 2. No | |  |
| Q205 | Does your family have any firm limits or agreements with the child about how much he/she can watch (TV, DVDs, electronic games, tablets, mobile/smart phone) or play video games? | | 1. Yes 2. No | |  |
| Q206 | If yes Q205, how much time the children are allowed to watch Television or play games per day? | | 1. 1-1:30  2. 1:30-2:30 | |  |
| Q207 | Do you soft drinks available in your home? | | 1. Yes 2. No | |  |
| Q208 | At home, does your child snack (on chips, biscuits.) and or drink soft drink whenever they like? | | 1. Yes 2. No | |  |
| Q209 | How often do parents offer your child sweets (cream cake, ice cream, cake, biscuits) to your child as a reward for good behavior? | | 1.Never  2.Sometimes  3. Usually | |  |
| Q210 | How much do you keep track of the sweets (candy, ice cream cake, pies, Pastries) that your child eats? | | 1.Never  2.Sometimes  3. Usually | |  |
| Q211 | How much do you keep track of the snack food (potato chips, meat, cheese puffs) that your child eats? | | 1.Never  2.Sometimes  3. Usually | |  |
| Q212 | How much do you keep track of the high fat foods that your child eats? | | 1.Never  2.Sometimes  3. Usually | |  |

**Verbal assent form for students**

Good morning/ afternoon?

My name is _____________________. Currently I am a graduate student at Debre Berhan University, Asrat Woldeyes Health science Campus, School of Nursing and Midwifery , Department of pediatrics Nursing and now I am conducting a research on prevalence of obesity and associated factors among school age children in Debre Berhan city.

**Objectives**: To assess prevalence and associated factors of obesity among school age children in Debre Berhan City, Ethiopia, 2022.

You are selected randomly as a possible participant in this study as a subject. I would like to ask you a few questions about your personal characteristics; your dietary habit, and your physical exercise, your willingness for weight and height measurements.

**Potential risks**: There is no potential risk that may cause any harm on study participants.

**Benefits:** No financial benefits are related with this study. But by participating in this study, you contribute to improve the prevention and control method of childhood obesity.

**Confidentiality:** You and your family name will not be written in this form and will never be used in connection with any information you tell us. All information given by you and your family will be kept strictly confidential. Your participation is voluntary and you are not obligate to answer any question which you do not wish to answer. If you fill discomfort to respond to the questioner, please fill free to drop it.

This interview will take about 30 minutes and weight and height measurement will be taken.

Interviewer sign ________________ date _______________code_____________

**Contact Address of the principal investigator**

Name: Abebe Nigussie Ayele

Email: [abebe2014nigussie@gmail.com](mailto:abebe2014nigussie@gmail.com)

Cell-phone: +251-920-633691/ 0953469427

Address**:** Debre Berhan

**PART II: Questionnaire for obesity and associated Factor for students**

Participant code ____________ Kebele------------- Name of the school --------------------

| **Socio-demography of the child, Location and type of school** | | | |
| --- | --- | --- | --- |
| Question | | Response | Remark |
| Q301 | Sex | 1. Female 2. Male |  |
| Q302 | Age | **-----------------------** |  |
| Q303 | Type of school | 1.Government 2.private |  |
| Q304 | Grade | ------------------- |  |

**Dieting habit-** The next questions ask about your dieting habit for the last one year.

| Question | | Response | Remark |
| --- | --- | --- | --- |
| Q401 | In a typical week on how many days do you eat fruit? | ------------ |  |
| Q402 | How many serving of fruit do you eat on one of those days? | --------------- |  |
| Q403 | In a typical week on how many days do you eat vegetables? | ----------------------- |  |
| Q404 | How many servings of vegetables do you eat on one of those days? | -------------------- |  |
| Q405 | Do you ever have a snack? | 1.Yes 2.No | If no got Q 407 |
| Q406 | How many times a day do you have snack? | ------------------- |  |
| Q407 | How many meal do you have a day other than snacks? | -------------------- |  |
| Q408 | How do you get your lunch? | 1. Bring from home  2. Buy from school cafeteria  3. Buy from nearby food service establishment.  4. I did not use lunch |  |
| Q409 | Foods that you ever bought in addition to the regular meal | 1.Cake  2.Biscuit  3.Ice cream  4.Chocolate  5.Others specify |  |
| Q410 | Do you eat While you Watch television? | 1. Yes 2. No  3.I did not watch television |  |
| Q411 | Do you eat your breakfast irregularly? | 1.Yes 2.No |  |
| Q412 | When you study do you eat food? | 1.Yes 2.No |  |

**Physical activity of the child -** Next I am going to ask you about time you spend doing different physical activities in a typical week.

| **Question** | | | | | | | Response | Remark | | |
| --- | --- | --- | --- | --- | --- | --- | --- | --- | --- | --- |
| Q501 | | Do you engaged in Work besides your education? | | | | | 1.Yes 2.No |  | | |
| Q502 | | If your answer in Q 501 is yes, does your work involve vigorous intensity activity that for at least 10 minutes continuously? | | | | | 1. Yes 2. No | if no go to Q505 | | |
| Q503 | | In a typical week on how many days do you do vigorous intensity activities as part of your work? | | | | | Number of days ---------- |  | | |
| Q504 | | How much time do you spend doing vigorous intensity activities at work on a typical day? | | | | | Hours: minutes ------- |  | | |
| Q505 | | Does your work involve moderate- intensity activity that causes small increases in breathing or heart rate for at least 10 minutes continuously? | | | | | 1. Yes 2. No | if no go to Q508 | | |
| Q506 | | In a typical week on how many day do you do moderate intensity? | | | | | Numbers of days------ |  | | |
| Q507 | | How much time do you spend doing moderate intensity activities at work on a typical day? | | | | | Hours: minutes -------- |  | | |
| Q508 | | Do you walk or use a bicycle for at least 10 minutes continuously to get to and from places? | | | | | 1.Yes 2.No | If no go to Q 511 | | |
| Q509 | | In a typical week on how many days do you walk or use a bicycle for at least 10 minutes continuously? | | | | | Numbers of day------ |  | | |
| Q510 | | How much time do you spend walking or bicycling for travel in a typical day? | | | | | Hours : minutes------ |  | | |
| Q511 | | Do you do any vigorous-intensity sports for at least 10 minutes continuously? | | | | | 1.Yes 2.No | if no got to Q514 | | |
| Q512 | | In a typical week on how many days do you do vigorous –intensity sports, fitness activities? | | | | | Numbers of day-------- |  | | |
| Q513 | | How much time do you spend doing vigorous –intensity sports, fitness or recreational activities in a typical days? | | | | | Hours : minutes-------- |  | | |
| Q514 | | Do you do any moderate-intensity sports that cause small increases in breathing or heart rate for at least 10 minutes continuously? | | | | | 1.Yes 2.No | if no got to Q517 | | |
| Q515 | | In a typical week, on how many days do you do moderate intensity sports, fitness or recreational (leisure) activities? | | | | | Numbers of day ------- |  | | |
| Q516 | | How much time do you spend doing moderate intensity sports, fitness or recreational activities in a typical days? | | | | | Hours : minutes------- |  | | |
| **Sedentary behavior** | | | | | | | | | | |
| Q517 | How do you spend your free time? | | | | | 1. Face book/telegram  2. Watching TV/ Video/film  3. Playing on computer games  4. Others( Specify)_______ | | | |  |
| Q518 | How much time do you usually spend on playing computer game/video game in a day? | | | | | Hours: minutes ----------- | | | |  |
| Q519 | How much time do you usually spend on Face book/telegram in a day? | | | | | Hours: minutes ----------- | | | |  |
| Q520 | How much time do you spend watching Television programs /film in a day? | | | | | Hours: minutes ----------- | | | |  |
| **Psychological factors** | | | | | | | | | | |
| **Sleep duration** | | | | | | | | | | |
| Q601 | | | Average Sleep duration in a day? | | Hours -------------- | | | | |  |
| Q602 | | | Do you have usually nap afternoon during a day? | | Hours :minutes------------ | | | | |  |
| **Loneliness –By UCLA loneliness scale** | | | | | | | | | | |
| Q603 | | | How often do you feel unhappy doing so many things alone? | | 1=Never 2=Rarely 3=Sometimes 4=Always | | | | |  |
| Q604 | | | How often do you feel you have no one to talk to? | | 1=Never 2=Rarely 3=Sometimes 4=Always | | | | |  |
| Q605 | | | How often do you feel you cannot tolerate being so alone? | | 1=Never 2=Rarely 3=Sometimes 4=Always | | | | |  |
| Q606 | | | How often do you feel as if no one understands you? | | 1=Never 2=Rarely 3=Sometimes 4=Always | | | | |  |
| Q607 | | | How often do you find yourself waiting for people to call or write? | | 1=Never 2=Rarely 3=Sometimes 4=Always | | | | |  |
| Q608 | | | How often do you feel completely alone? | | 1=Never 2=Rarely 3=Sometimes 4=Always | | | | |  |
| Q609 | | | How often do you feel unable to reach out and communicate with those around you? | | 1=Never 2=Rarely 3=Sometimes 4=Always | | | | |  |
| Q610 | | | How often do you feel alone in your school? | | 1=Never 2=Rarely 3=Sometimes 4=Always | | | | |  |
| Q611 | | | How often do you feel it is difficult for you to make friends? | | 1=Never 2=Rarely 3=Sometimes 4=Always | | | | |  |
| Q612 | | | How often do you feel shut out and excluded by others? | | 1=Never 2=Rarely 3=Sometimes 4=Always | | | | |  |
| Q613 | | | Do you have close friends in school or neighbors? | | 1. Yes 2. No | | | | |  |
| **Part III:** **Questionnaire for obesity and associated Factor Surveillance for Physical Measurements to be filled by data collectors** | | | | | | | | | | |
| **Measurement** | | | | Reading | | | | | Remark | |
| Q701 | | | Height | In centimeters ----------- | | | | |  | |
| Q702 | | | Weight | In kilograms (Kg)-------- | | | | |  | |
