# Supplementary material for: Predictors of obesity among school-age children in Debre Berhan City, Ethiopia
Source: PLOS Glob Public Health. 2023 Sep 8;3(9):e0001895. doi: 10.1371/journal.pgph.0001895 (PMC10490846; doi:10.1371/journal.pgph.0001895)
Supplement: Attachment — Submitted filename: Response to Reviewers.docx [file pgph.0001895.s003.docx]

Letter of response

**To all honourable reviewers and editors**

Dear Reviewers and Editors, We are very appreciative for your relevant comments, questions and recommendations. Following this, we have provided our responses point by point as presented below.

**Authors Responses**
1. Does this manuscript meet PLOS Global Public Health’s publication criteria? Is the manuscript technically sound, and do the data support the conclusions? The manuscript must describe methodologically and ethically rigorous research with conclusions that are appropriately drawn based on the data presented.

**Reviewer #**1: Yes

N/A

2. Has the statistical analysis been performed appropriately and rigorously?

**Reviewer #1**: Yes

N/A

3. Have the authors made all data underlying the findings in their manuscript fully available (please refer to the Data Availability Statement at the start of the manuscript PDF file)?

The PLOS Data policy requires authors to make all data underlying the findings described in their manuscript fully available without restriction, with rare exception. The data should be provided as part of the manuscript or its supporting information, or deposited to a public repository. For example, in addition to summary statistics, the data points behind means, medians and variance measures should be available. If there are restrictions on publicly sharing data—e.g. participant privacy or use of data from a third party—those must be specified.

**Reviewer #1**: No

**Answer**:

Thanks to your constructive comments and suggestions, now we all the authors accept amend it accordingly.

4. Is the manuscript presented in an intelligible fashion and written in Standard English?

PLOS Global Public Health does not copyedit accepted manuscripts, so the language in submitted articles must be clear, correct, and unambiguous. Any typographical or grammatical errors should be corrected at revision, so please note any specific errors here.

**Reviewer #1**: Yes

N/A

5. Review Comments to the Author

Please use the space provided to explain your answers to the questions above. You may also include additional comments for the author, including concerns about dual publication, research ethics, or publication ethics. (Please upload your review as an attachment if it exceeds 20,000 characters)

**Reviewer #1**: 1. Authors give information about mothers' age. But there are no information about fathers' age.

**Answer**:

His Excellency, you are write and we recommend it for the future researchers to incorporate it and we state it as a limitation.

2. Figures 1-3 do not have any new research information. I recommend to reduce these tables.

**Answer**:

We accept and amended it accordingly.

3. In discussion authors have to add: what to do? For example, life skills lessons might be organized in the school. Some unformal education.... and so on

**Answer**:

Thanks to your agreeable comments and we have been accepted your suggestion and try to add some ideas what to the responsible body for each sectio.

6. PLOS authors have the option to publish the peer review history of their article (what does this mean?). If published, this will include your full peer review and any attached files.

**Answer:**

We all accepted the suggestion raised by your amended it on the dashboard to disclose the peer review document.

Do you want your identity to be public for this peer review? If you choose “no”, your identity will remain anonymous but your review may still be made public.

**Answer**:

**We amended it (Yes) and to disclose**

For information about this choice, including consent withdrawal, please see our Privacy Policy.

Reviewer #1: No

**Answer**:

**We have been amended it.**

Hanna Nalecz, Ph.D.

Academic Editor

PLOS Global Public Health

Journal Requirements:

1. Please review your reference list to ensure that it is complete and correct. If you have cited papers that have been retracted, please include the rationale for doing so in the manuscript text, or remove these references and replace them with relevant current references. Any changes to the reference list should be mentioned in the rebuttal letter that accompanies your revised manuscript. If you need to cite a retracted article, indicate the article’s retracted status in the References list and also include a citation and full reference for the retraction notice.

Answer:

N/A

2. Please send a completed 'Competing Interests' statement; including any COIs declared by your co-authors. If you have no competing interests to declare, please state "The authors have declared that no competing interests exist". Otherwise please declare all competing interests beginning with the statement "I have read the journal's policy and the authors of this manuscript have the following competing interests:"

Answer:

We accepted and amended it accordingly

3. Please provide separate figure files in .tif or .eps format only and remove any figures embedded in your manuscript file. Please also ensure that all files are under our size limit of 10MB.

Answer:

We accepted and amended it accordingly

4. If you did not receive any funding for this study, please simply state: “The authors received no specific funding for this work.”

Answer:

We accepted and amended it accordingly

5. Please ensure that all Figure files have corresponding citations and legends within the manuscript. Currently, Figures 1-3 in your submission file inventory does not have an in-text citation. If the figure is no longer to be included as part of the submission, please remove it from the file inventory.

Answer:

We accepted comments and amended it accordingly

6. Please provide a complete Data Availability Statement in the submission form, ensuring you include all necessary access information or a reason for why you are unable to make your data freely accessible. If your research concerns only data provided within your submission, please write "All data are in the manuscript and/or supporting information files" as your Data Availability Statement.

Answer:

We accepted and amended it accordingly on the Data Availability Statement submission form.

*We thanks to much for your quick response!*
