# Supplementary material for: Predictors of obesity among school-age children in Debre Berhan City, Ethiopia
Source: PLOS Glob Public Health. 2023 Sep 8;3(9):e0001895. doi: 10.1371/journal.pgph.0001895 (PMC10490846; doi:10.1371/journal.pgph.0001895)
Supplement: Attachment — Submitted filename: Response to Reviewers.docx [file pgph.0001895.s004.docx]

**Letter of response**

**To all honourable reviewers and editors**

Dear Reviewers and Editors, We are very appreciative for your relevant comments, questions and recommendations. Following this, we have provided our responses point by point as presented below.

**Authors Responses**
1. If the authors have adequately addressed your comments raised in a previous round of review and you feel that this manuscript is now acceptable for publication, you may indicate that here to bypass the “Comments to the Author” section, enter your conflict of interest statement in the “Confidential to Editor” section, and submit your "Accept" recommendation.

**Reviewer #2**: All comments have been addressed

N/A

2. Does this manuscript meet PLOS Global Public Health’s publication criteria? Is the manuscript technically sound, and do the data support the conclusions? The manuscript must describe methodologically and ethically rigorous research with conclusions that are appropriately drawn based on the data presented.

**Reviewer #2:** Yes

N/A

3. Has the statistical analysis been performed appropriately and rigorously?

Reviewer #2: Yes

N/A

4. Have the authors made all data underlying the findings in their manuscript fully available (please refer to the Data Availability Statement at the start of the manuscript PDF file)?

The PLOS Data policy requires authors to make all data underlying the findings described in their manuscript fully available without restriction, with rare exception. The data should be provided as part of the manuscript or its supporting information, or deposited to a public repository. For example, in addition to summary statistics, the data points behind means, medians and variance measures should be available. If there are restrictions on publicly sharing data e.g. participant privacy or use of data from a third party those must be specified.

Reviewer #2: Yes

N/A

5. Is the manuscript presented in an intelligible fashion and written in Standard English?

PLOS Global Public Health does not copyedit accepted manuscripts, so the language in submitted articles must be clear, correct, and unambiguous. Any typographical or grammatical errors should be corrected at revision, so please note any specific errors here.

Reviewer #2: Yes

N/A

6. Review Comments to the Author

Please use the space provided to explain your answers to the questions above. You may also include additional comments for the author, including concerns about dual publication, research ethics, or publication ethics. (Please upload your review as an attachment if it exceeds 20,000 characters)

Reviewer #2: The title require modifications: need to remove redundant words and align with statistical analysis involved, thus; "Predictors of Obesity among School-Age Children in Debre Berhan City, Ethiopia" The phrase "...-A Cross-Sectional Study" is redundant and has no function since the title expresses itself as an observational study seeking to identify predictors of obesity. With these there should be alignment between the title and the data analysed.

**Answer**:

We accept and amended it accordingly.

7. PLOS authors have the option to publish the peer review history of their article (what does this mean?). If published, this will include your full peer review and any attached files.

Do you want your identity to be public for this peer review? If you choose “no”, your identity will remain anonymous but your review may still be made public.

Answer

We accept and do it

For information about this choice, including consent withdrawal, please see our Privacy Policy.

Reviewer #2: Yes: Nnodimele Onuigbo ATULOMAH

N/A

Hanna Nalecz, Ph.D.

Academic Editor

PLOS Global Public Health

*We thanks to much for your quick response!!!!!!!*
